# The endophytic microbiome response patterns of *Juglans regia* to two pathogenic fungi

**DOI:** 10.3389/fmicb.2024.1378273

**Published:** 2024-04-11

**Authors:** Ziye Wang, Lu Xu, Xiaoyue Lu, Ruidong Wang, Jie Han, Aihua Yan

**Affiliations:** ^1^Key Laboratory of Forest Protection of National Forestry and Grassland Administration, Chinese Academy of Forestry, Ecology and Nature Conservation Institute, Beijing, China; ^2^College of Forestry, Hebei Agricultural University, Baoding, Hebei, China; ^3^Hebei Province Key Laboratory of Forest Trees Germplasm Resources and Forest Protection, Baoding, Hebei, China; ^4^Hebei Urban Forest Health Technology Innovation Center, Baoding, Hebei, China

**Keywords:** endophytic microbiome, pathogenic fungi, metabolome, endogenous antagonistic bacteria, expression mode

## Abstract

The endophytic microbial community reassembles to participate in plant immune balance when the host plants are stressed by pathogens. However, it remains unclear whether this assembly is pathogen-specific and how regulatory pathways are coordinated in multi-pathogens. In order to investigate the effects of infection with *Colletotrichum gloeosporioides* (Cg treatment) and *Fusarium proliferatum* (Fp treatment) on walnut leaf endophytic microbiome in their assembly, co-occurrence pattern, and on comprehensive chemical function of the internal environment of leaf, an interaction system of the walnut–pathogenic fungi was constructed using seed embryo tissue culture technology. The study showed differences in the assembly of endophytic microbial communities in walnut trees across three groups (control group, Ck; Cg; Fp) after Cg and Fp treatments. Despite changes in relative abundances, the dominant communities in phyla and genera remained comparable during the infection of the two pathogens. Endophyte fungi were more sensitive to the pathogen challenge than endophyte bacteria. Both promoted the enrichment of beneficial bacteria such as *Bacillus* and *Pseudomonas*, changed the modularity of the community, and reduced the stability and complexity of the endophyte community. Pathogenic fungi infection mainly affects the metabolism of porphyrin and chlorophyll, purine metabolism, phenylpropane metabolism, and amino acid metabolism. However, there was no significant difference in the secondary metabolites for the different susceptible plants. By screening endogenous antagonistic bacteria, we further verified that *Pseudomonas psychrotolerans* and *Bacillus subtilis* had inhibitory effects on the two pathogenic fungi and participated in the interaction between the leaves and pathogenic fungi. The antibacterial substances may be 1-methylnaphthalene, 1,3-butadiene, 2,3-butanediol, and toluene aldehyde.

## 1 Introduction

*Juglans regia*, also known as the walnut tree, holds great economic and ecological value in China due to its woody grain and oil (Su et al., 2020). However, this species faces significant challenges from pathogenic fungi, specifically *Colletotrichum gloeosporioides* and *Fusarium proliferatum* (Crous et al., 2004; Wang et al., 2022), which can cause leaf spot. During the initial stages of the disease, irregular brown spots appear on the leaf edges, causing them to shrivel. In later stages, the disease progresses inward along the edges, affecting the entire leaves, which then wither (Wang et al., 2022). Currently, endophytes are being proposed as a new research idea for regulating plant diseases. Endophytes colonize plant cells and intercellular spaces without causing plant pathogenesis. They often provide essential nutrients (Lu et al., 2018), promote growth (Chialva et al., 2018; Dubey et al., 2019), and enhance stress tolerance (Berendsen et al., 2012). Therefore, endophytes are an important area of research for understanding plant defense mechanisms and promoting sustainable agricultural practices.

Pathogens trigger the plant immune response of microbe-associated molecular patterns (MAMPs) through correlated signaling pathways to further regulate the host–microbiome association network (Pieterse et al., 2014). Endophytic communities contain specific functional groups that enhance plant disease resistance, while others may act as latent pathogenic elements ready to proliferate (Cordovez et al., 2019). Competitive interactions between different functional microbial groups primarily influence the colonization or expansion of pathogens (Berendsen et al., 2018; Liu et al., 2020). Bioactive metabolites in diseased plants have been classified into various structural groups, including alkaloids, terpenoids, steroids, quinones, phenols, coumarins, glycosides, and benzopyrones as shown by metabolomic analyses (Zhang et al., 2006; Gouda et al., 2016). Endophytic bacteria and host plants may produce these metabolites separately or together, with different metabolic functions and interactions. Elucidating these mechanisms and identifying key microbial players that can be exploited for biocontrol strategies should be the focus of research (Spadaro and Droby, 2016). The metabolites of plant–endophytic microbial community interactions should be further systematically investigated to understand the role of such interactions in signaling crosstalk that facilitates plant growth, their role in stress regulation and to provide new insights for plant-wide biotic call-and-rescue strategies (Noecker et al., 2019; Li et al., 2022).

Endophytic microbial communities play a crucial role in plant growth and immune homeostasis. They are reconstituted when plants are stressed by pathogens. This study utilized seed embryo tissue culture technology to establish a mutualistic walnut–pathogenic fungal system. The microbial communities and non-targeted metabolomic assays were systematically analyzed to determine changes in species diversity of the endophytic leaf microbiome and the responses of walnuts to different pathogenic fungal stresses. We investigated functional core groups with broad-spectrum resistance and analyzed the effects of two pathogenic fungi on the aggregation and symbiosis patterns of endophytic microorganisms in leaves. This study provides a theoretical basis for developing microecological control technologies and products for the integrated prevention and control of various walnut diseases.

## 2 Materials and methods

### 2.1 Sample treatment and collection

Walnut seeds were selected from the “LVLING” fruit harvested by the walnut demonstration garden base of Hebei Lyuling Company, Lincheng County. Walnut seed embryo plant tissue culture seedlings of uniform growth, approximately 15 cm in height, were selected. We used inoculated sterile water as a control group (CK). The treatment groups were inoculated with pathogenic fungal (*C. gloeosporioides* [Cg] and *F. proliferatum* [Fp]) spore suspensions (concentration: 1.8 × 10^7^ units/mL). A sterile 0.5 cm × 0.5 cm piece of filter paper was used to suck up the spore suspension or sterile water to cover the surface of the leaf. The leaves were collected separately under aseptic conditions 7 days after inoculation. They were placed in sterile centrifuge tubes, flash froze with liquid nitrogen, and stored at −80°C. Samples were subsequently packed with dry ice and shipped to Beijing Biomarker Technologies for high-throughput sequencing.

### 2.2 Microbiome extraction and data analysis

Sample DNA was extracted using the TGuide S96 Magnetic Universal DNA Kit (Tiangen Biochemical Technology (Beijing) Co.). Endophytic bacterial DNA was amplified using universal primers 338F/806R for the 16S V3-V4 region, and the endophytic fungi were amplified using ITS1F/ITS2R (Zheng et al., 2015). The PCR amplification reaction program parameters were as follows: pre-denaturation at 95°C for 5 min; denaturation at 95°C for 30 s, annealing at 50 for 30 s, and extension at 72°C for 40 s for 30 cycles; and finally extension at 72°C for 7 min. The PCR products were tested for integrity by electrophoresis on a 1.8% (w/v) agarose gel.

The constructed libraries were sequenced using Illumina NovaSeq6000 (Illumina). The raw sequences (raw reads) obtained from sequencing were screened, analyzed, and discarded using Cutadapt software (Cutadapt 1.9.1) to obtain clean reads without primer sequences. Double-end sequence splicing was performed using USEARCH v10 software to overlap the clean reads of each sample. Effective reads were obtained after identifying and eliminating chimeric sequences.

The clean data were clustered into OTUs and assigned to taxonomic lineages for each sample. The alpha diversity indices of endophytic bacterial and fungal communities in different treatment groups, Chao1 and Shannon, were calculated using QIIME2 2020.6 (Bolyen et al., 2019) software, and the significance of differences was verified using Student’s *t*-test. Beta-diversity analysis was performed using QIIME software to compare the similarity of different samples in terms of species diversity. The distance between samples was calculated using Bray–Curtis to obtain beta values between samples. These values were then visualized on a non-metric multidimensional scaling (NMDS) chart (Looft et al., 2012). The main distribution characteristics of the microbial communities in the samples were obtained after statistical analysis (Schloss et al., 2009). Based on the abundance and variation of each species in each sample, Spearman’s rank correlation analysis was performed and the correlation network was constructed by filtering the data with a correlation greater than 0.1 and *p*-value less than 0.05, and the correlation network of the genera with a relative abundance of top 80 was visualized using Gephi 0.10.

### 2.3 Metabolite extraction and data analysis

For metabolite extraction, 1,000 μL of extraction solution (methanol: acetonitrile: water = 2:2:1; internal standard concentration: 2 mg/L) containing an internal standard (1,000, 2, concentration: 2 mg/L) was added to a 50-mg leaf sample. The solution was stirred for 30 s and then pulverized for 10 min in a 45-Hz grinder, followed by 10 min of ultrasonication in an ice bath. The samples were then allowed to rest for 1 h at −20°C, succeeded by centrifugation for 15 min at 12000 rpm and 4°C. The supernatant (500 μL) was then dried under vacuum. A volume of 160 μL of the extract (acetonitrile to water 1:1) was redissolved, centrifuged for 30 s, soaked in ice water for 10 min, and finally centrifuged at 12000 rpm for 15 min at 4°C. The supernatant (120 μL) was pipetted as the assay sample, and a certain amount of the supernatant was reserved from each sample. The reserved supernatant of each sample was injected into an LC–MS for analysis (Want et al., 2010; Dunn et al., 2011).

Peak extraction and alignment data (Progenesis QI software) were processed, and mass number deviation (< 100 ppm) was controlled and normalized (Wang et al., 2016). Metabolites were classified and searched in the Kyoto Encyclopedia of Genes and Genomes (KEGG) databases for qualitative and quantitative purposes (Kanehisa and Goto, 2000; Yu et al., 2012). Tests of variance were performed for each treatment group (fold change [FC] > 1, the *t*-test *p*-value <0.01, and OPLS-DA model VIP > 1). The differential metabolites were screened by combining the FC value, the *t*-test *p*-value, and the VIP value of the OPLS-DA model. The screening criteria were as follows: FC > 1, *p*-value <0.05, and VIP > 1 (FC > 2: upregulation; FC < 1/2: downregulation; FC = 1: screening for differences was equivalent to disregarding FC).

### 2.4 Determination of the antagonistic effect of endophytic bacteria

Walnut leaves were rinsed under running tap water to remove impurities from the leaf surface, and the rinsed leaves were placed in an ultraclean bench to sterilize the leaf surface: The leaf surface was rinsed with sterile water and then vacuumed to dry the residue of sterile water on the surface, followed by gradual sterilization using 75% alcohol (5–10 s), 0.1% mercuric chloride solution (5 min), and finally, the leaf surface was rinsed again with sterile water for five times. Walnut leaves were ground into a homogenate in a sterile mortar, and the homogenate was placed in a triangular flask containing 90 mL of sterile water. A certain amount of fetching dilution was evenly spread on the sterile LB plate medium, and three replicates were set up for the control and the treatment groups, respectively, which were inverted and cultivated in an artificial light incubator at a temperature of 28°C. The growth of the colonies was checked at any time, and the single colonies were further used for purification culture (Zheng et al., 2021).

The inhibition capacity of the strain in the fermentation broth was determined using the endophytic bacteria isolated by the coated plate method (Wang et al., 2011) and the spore germination method (Fang, 1979). Spore germination rate, mycelial growth inhibition rate, incidence rate, disease index, and control efficacy were counted, and the significance of differences was calculated using one-way ANOVA. Subsequently, isolated and living leaves were used to further determine the bacterial inhibitory ability of the strains. 7d-activated pathogenic fungi were made into disks (diameter: 0.5 cm) using a perforator; the configured bacterial fungal solution was sprayed on the surface of the leaves, and the leaves were well moisturized; and the incidence and disease indices, as well as the bacterial efficacy in controlling the pathogenic fungi, were calculated for each treatment after 9 d (Fang, 1979).

Strains with antagonistic effects were subjected to 16S rDNA sequencing using primers 27F 5’-AGAGTTTGATCCTGGCTCAG-3′ and 1492R 5’-CTACGGCTACCTTGTTACGA-3′. The quality of amplification products was tested and sequence detection was performed to compare sequence similarity (GenBank platform); the phylogeny of the sequences was analyzed using the adjacent distance method in MEGA 7.0 software, and a phylogenetic tree was constructed to draw the phylogenetic tree.

Expression of antagonistic bacteria within leaves at different times was analyzed using real-time fluorescence quantification. The metabolites of the strain were determined using liquid mass spectrometry (MS) analysis. Data were processed using Excel 2003, SPSS version 26.0 software for one-way ANOVA, Duncan’s multiple range test (MRT) to test for significant differences (*p* < 0.05), and Origin 2021 software to construct graphs.

## 3 Results and analysis

### 3.1 Endophytic microbial community assembly

#### 3.1.1 Endophytic microbial diversity analysis

**Figure 1:**
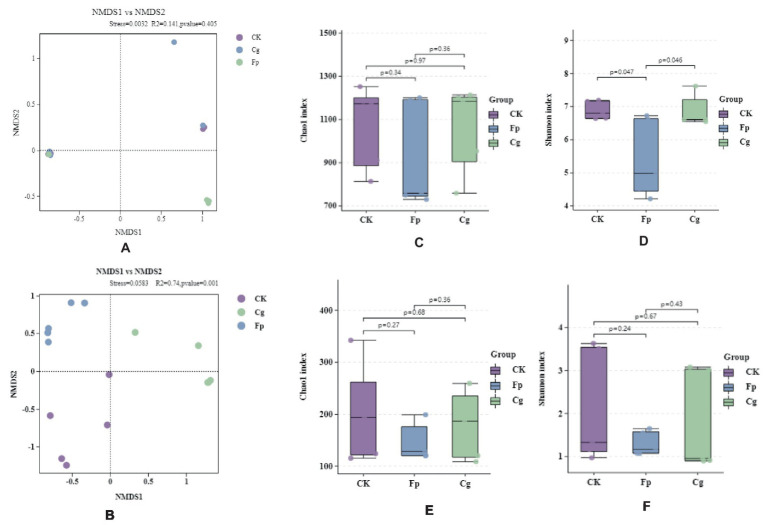
Endophytic microbial community diversity. **(A,B)** Endophytic bacterial and fungal β-diversity, respectively; **(C–F)** Endophytic microbial community α-diversity; **(C,E)** Chao1 index for endophytic bacterial and fungal communities, respectively; **(D,F)** Shannon index for endophytic bacterial and fungal communities, respectively.

After infection with *C. gloeosporioides* and *F. proliferatum*, between groups were greater than the differences within groups Infection in significant differences in endophytic fungal communities, and non-significant differences in endophytic bacterial communities (Figures 1A,B). The Chao1 and Shannon diversity indices showed that the diversity of endophytic bacteria was slightly lower after pathogenic fungal infection, although the difference was not statistically significant (*p* > 0.05). The effect of Cg infection on endophytic bacterial richness was small, but Fp infection significantly reduced endophytic bacterial richness (Figures 1C,D). Pathogenic bacterial infection reduced the abundance and diversity of endophytic fungal communities, although the difference was insignificant (*p* > 0.05). Endophytic fungal abundance and diversity were higher after Cg infection than after Fp infection (Figures 1E,F).

#### 3.1.2 Endophytic microbial community composition

**Figure 2:**
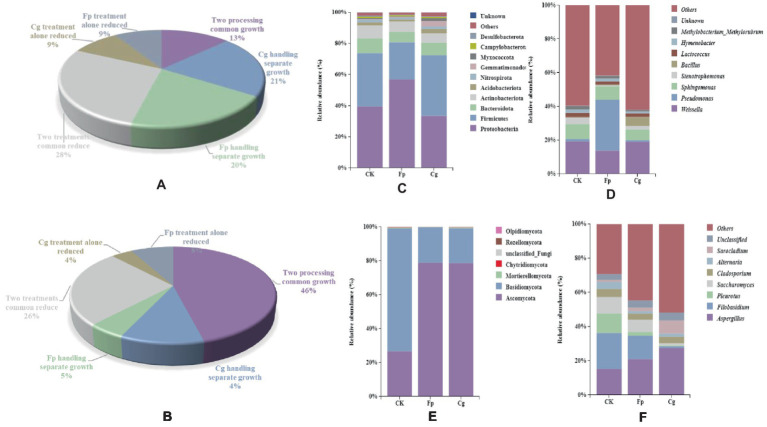
Species composition of endosymbiotic communities. **(A,B)** Statistics of relative abundance changes in endophytic bacterial and fungal communities, respectively; **(C,D)** Species composition of endophytic bacterial communities at the phylum and genus level; and **(E,F)** Species composition of endophytic fungal communities.

The endophytic microbial community showed an increase in the relative abundance of endophytic bacteria (13%) and endophytic fungi (46%) but also showed a decrease in the relative abundance of endophytic bacteria (28%) and endophytic fungi (26%). Thus, the endophytic microbial community simultaneously responded to the infection of both pathogenic fungi. In addition, we observed relative abundance changes in the endophytic species level in response to the infection of both pathogenic fungi (Figures 2A,B).

The dominant phylum of the endophytic microbiome had the same genus but differed in relative abundance. The dominant phyla for the endophytic bacterial community were Proteobacteria, Firmicutes, Bacteroidetes, and Actinobacteria, and the top five dominant genera included *Weissella*, *Pseudomonas*, *Sphingomonas*, *Stenotrophomonas*, and *Bacillus*. The relative abundance of *Bacillus* increased in response to both pathogens. In addition, the relative abundance of *Weissella* increased due to Cg infection, while the relative abundance of *Pseudomonas* increased by Fp infection. The relative abundance of *Bacillus* and *Pseudomonas* significantly differed between treatments (Figures 2C,D). The dominant phyla in the endophytic fungal community were Ascomycota, Basidiomycota, and Mortierellomycota. The pathogenic fungal infection increased the relative abundance of Ascomycota and decreased the relative abundance of Stenotrophomonas. The dominant genera included *Aspergillus*, *Filobasidium*, and *Pleurotus*. The pathogenic fungal infection reduced the relative abundance of the potential pathogenic fungi *Aspergillus*, *Filobasidium*, and *Pleurotus* (Figures 2E,F).

#### 3.1.3 Endophytic microbial community co-occurrence network

**Figure 3:**
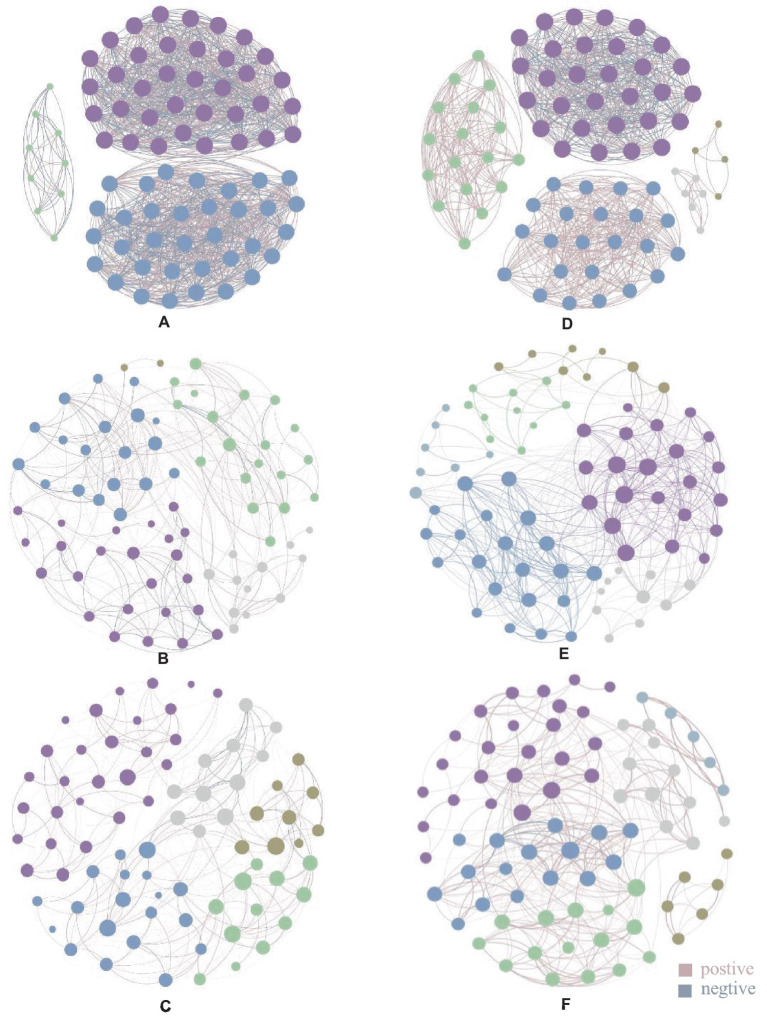
Endophytic microbial community association network diagram. Panels **(A-C)** are endophytic bacterial communities, panel **(A)** is CK endophytic bacterial community, panel **(B)** is endophytic bacterial community after Cg treatment, and panel **(C)** is endophytic bacterial community after Fp treatment; panel **(D-F)** are endophytic fungal communities, panel **(D)** is CK endophytic fungal community, panel **(E)** is endophytic fungal community after Cg treatment, and panel **(F)** is endophytic fungal community after Fp treatment. Different colored nodes in the diagram indicate different modules, and the lines between the dots indicate the correlation between the two nodes, with pink lines indicating positive correlation and gray lines indicating negative correlation; the thickness of the lines indicates the level of correlation.

In the endophytic bacterial network, the top 80 genera with the highest relative abundance were selected to construct the co-occurrence network map. The control group (CK) had 1,227 edges, 79 nodes 3 modules, and 49.47% of the negative correlation (Supplementary Table S1). *Weissella* had 35 associations with genera with the highest relative abundance and 18 negative associations (Supplementary Table S2). The Cg treatment group had 369 edges, 76 nodes, 5 modules, and 32.79% of the negative correlation (Supplementary Table S1). *Weissella* has fewer associations than CK. *[Ruminococcus]_torques_group* has the largest number of associations (Supplementary Table S3). The Fp treatment group had 327 edges, 79 nodes, 5 modules, and 10.4% of the negative correlation (Supplementary Table S1). *Weissella* has fewer associations than CK. *Staphylococci* have the largest number of associations (Supplementary Table S4). After the infection of two pathogenic fungi, the modularity of the endophytic bacterial network was changed, the correlation of the network was reduced, and the proportion of negative correlation was reduced, thus affecting the stability of the community (Figures 3A–C and Supplementary Figures S1–S3).

In the endophytic fungal association network, the top 80 genera with the highest relative abundance were selected to construct the map. As shown in Figures 3D–F, the control group (CK) produced 845 edges, 5 modules, and 29.11% of the negative correlation (Supplementary Table S1). *Filobasidium* had the highest relative abundance with 29 associated genera (Supplementary Table S5); 78 nodes, 471 edges, 6 modules, and 23.99% of the negative correlation were found in the network graph for the Cg treatment group (Supplementary Table S1). *Penicillium* had the highest relative abundance with 25 associated genera (Supplementary Table S6). The network diagram for the Fp treatment group had 78 nodes, 370 edges, 6 modules, and 5.14% of the negative correlation (Supplementary Table S1). *Alternaria* has the highest number of associations (Supplementary Table S7). The results showed that the number of modules in the endophytic fungal network increased and the proportion of associated edges and negative associations decreased after infection by the two pathogenic fungi (Figures 3D–F and Supplementary Figures S4–S6).

### 3.2 Plant differential metabolite screening

The positive ion model enriched for 1,549 metabolites and 115 secondary metabolites, including 160 amino acids, 18 indoles, 12 coumarins, 127 flavonoids, and 14 unsaturated fatty acids. Of these compounds, five amino acids were downregulated, three flavonoids were downregulated, four were upregulated, two indoles were upregulated, and one unsaturated fatty acid was upregulated. For the phytohormones, nine metabolites of ethylene, four metabolites of salicylic acid, and one metabolite of abscisic acid were identified.

**Figure 4:**
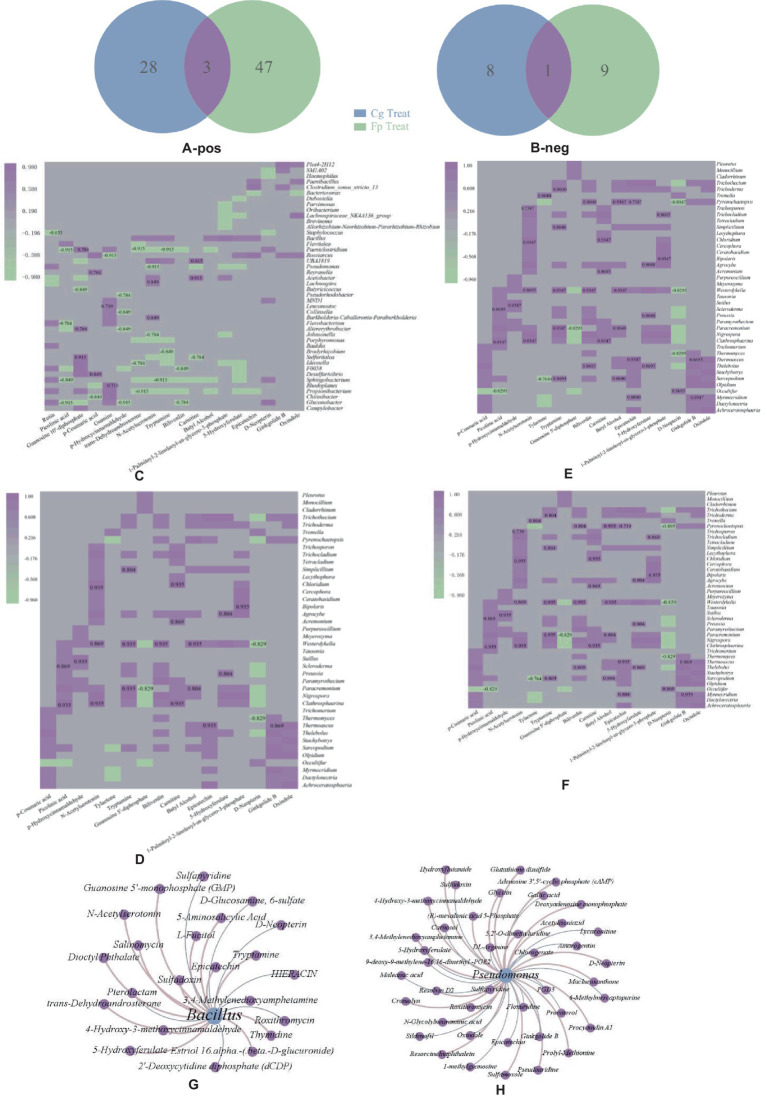
**(A,B)** Positive ion pattern differential metabolite Wein diagrams, respectively. Numbers in the graph are differential metabolite trees: blue letters are the same downregulated metabolites after two treatments; red letters are upregulated metabolites; **(C,D)** Heat maps of correlation between differential metabolites and co-variant endophytic bacteria; **(E,F)** Heat maps of correlations between differential metabolites and co-variant endophytic fungi; **(C,E)** Cg and Fp treatments, respectively. Horizontal coordinates are differential metabolites, and vertical coordinates are endophytic bacteria. The red box in the graph shows the increase in expression or relative abundance, and the blue box shows the decrease; **(G,H)** Association plots of the two endogenous antagonistic bacterial strains with differential metabolite networks.

A total of 134 differential metabolites were obtained between the groups, as shown in Figures 4A,B. There were eight identical differential metabolites after infection by the two pathogenic fungi, and biliverdin, tryptamine, and estradiol cypionate were significantly upregulated.

### 3.3 Combined microbiome and metabolome analysis

Endophytic microorganisms that co-responded to pathogenic fungal infection were correlated to differential metabolites annotated with the KEGG pathway. Heat maps were drawn using significantly correlated data (CCP < 0.05). As shown in Figures 4C,D, differential metabolites significantly correlated with a large number of co-variant endophytic bacteria after infection with both pathogenic fungi. The common differential metabolite, biliverdin, significantly negatively correlated with the endophytic bacteria *Clostridium paeoniae, Paeniclostridium*, *Aedonella* spp., and *Ideonella* after Cg and Fp infection. Tryptamine significantly negatively correlated with *Porphyromonas* spp. Guanosine 5′-diphosphate showed highly significant positive correlations with *Lactobacillus dubosiella*, *Ideonella*, and *Paeniclostridium* after Cg infection but also significantly positively correlated with *Alternaria alternata* and *Altererythrobacter*. Citrulline was highly significantly positively correlated with *Altererythrobacter* and *Ideonella* but also significantly positively correlated with *Dubosiella* and *Paeniclostridium* after Fp infection.

As shown in Figures 4E,F, the Cg treatment group was more highly correlated with co-variant endophytic fungi than the Fp group. The metabolite, biliverdin, highly significantly and positively correlated with the endophytic fungi *Clathrosphaerina*, *Nigrospora*, *Paracremonium*, and *Westerdykella* after Cg infection but did not show correlations for the Fp infection group. The common differential metabolite tryptamine significantly positively correlated with the endophytic fungi *Clathrosphaerina*, *Sarocladium*, *Simplicillium*, *Trichocladium*, *Trichoderma*, and *Trichothecium* after Cg and Fp infection. Guanosine 5′-diphosphate significantly positively correlated with the endophytic fungi *Clathrosphaerina* after Cg and Fp infection, respectively. There was no significant correlation with endophytic fungi after Fp infection.

#### 3.3.1 Endophytic microbiome: differential genera and metabolite associations

The differential endophytic bacteria, *Bacillus* spp., and *Pseudomonas* spp., were associated with 17 and 43 metabolites, respectively. In total, four and five metabolic pathways were enriched for *Bacillus* and *Pseudomonas*, respectively; one metabolite was upregulated, and three were concurrently downregulated by the two pathogenic fungi. D-neopterin was upregulated and is mainly involved in the folic acid biosynthesis of 3,4-methylenedioxyamphetamine. 3,4-Methylenedioxyamphetamine is involved in phenylpropanoid biosynthesis and is also associated with metabolites such as sulfapyridine and roxithromycin (Figures 4G,H).

### 3.4 Screening of endogenous antagonistic bacterial strains and identification of inhibition ability

**Figure 5:**
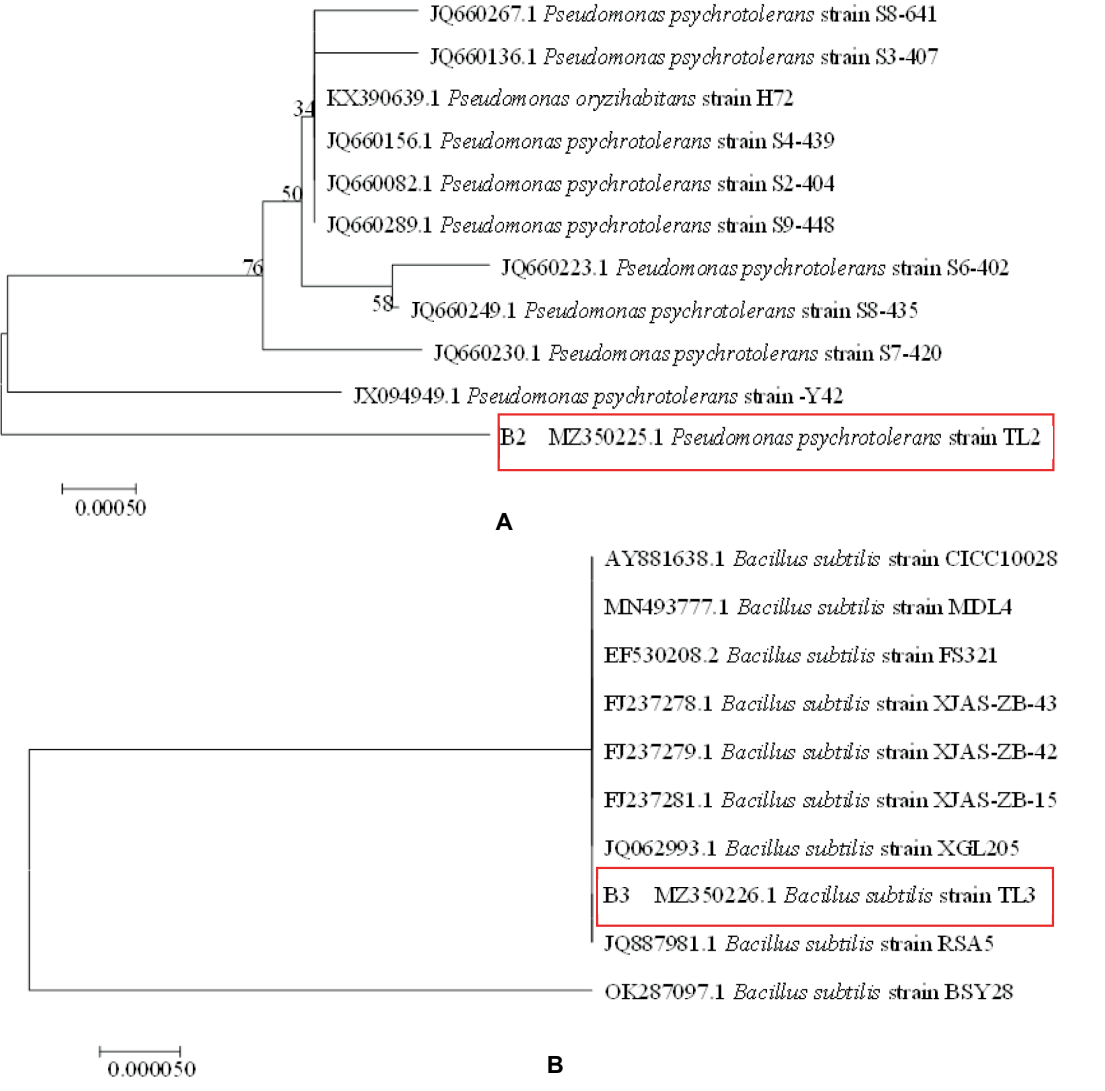
Phylogenetic tree of antimicrobial resistance: panel **(A)** is *Pseudomonas psychrotolerans*, panel **(B)** is *Bacillus subtilis*.

**Figure 6:**
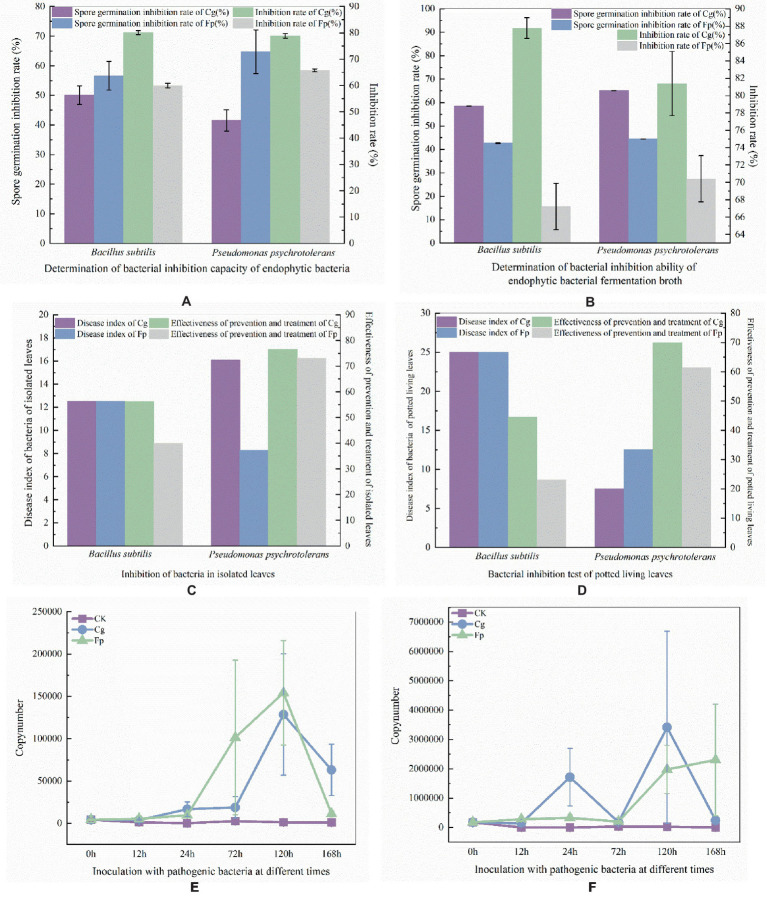
**(A)** Laboratory determination of antibacterial ability; **(B)** Determination of endophytic bacteria fermented liquid antibacterial ability (**(A,B)** the left axis of the coordinate axis is the rate of spore germination and the right axis is the rate of mycelial growth inhibition). **(C)** *In vitro* antibacterial ability; **(D)** Living blade bacteriostatic ability determination (**(C,B)** the left axis of the coordinates is the disease index and the right axis is the control effect). Panel **(E)** is expression of B. subtilis after infection by pathogenic fungi; Panel **(F)** is expression of *P. psychrotolerans* after infection by pathogenic fungi. Quantity determination of different time periods (**(E,F)** the vertical axis of the coordinates is the expression, and the horizontal axis is the different times of the treatment).

Pre-experimental screening of antagonistic bacteria yielded two strains with better inhibitory effects. The two strains of resistant bacteria were identified by molecular biology as *Pseudomonas psychrotolerans* and *Bacillus subtilis* (Figures 5A,B), which were isolated and screened. *P. psychrotolerans* inhibited the germination of Cg and Fp spores by 50.06 and 56.63%, respectively. *P. psychrotolerans* inhibited mycelial growth in the fermentation broth by inhibiting the germination of Cg and Fp spores by 41.54 and 64.75%, where the mycelial growth inhibition rates were 78.86 and 65.79%, respectively (Figure 6A). The *B. subtilis* fermentation broth inhibited the germination of Cg and Fp spores by 58.59 and 42.65%, and the mycelial inhibition rates were 87.78 and 67.22%, respectively (Figure 6B). The inhibition ability of the isolated leaves was as follows: Cg and Fp incidence decreased by 51.26 and 52.39% after the control of *P. psychrotolerans*, and the control effect for Cg and Fp was 76.58 and 73.12%, respectively. The Cg and Fp incidence decreased by 44.12 and 35.71% after the control of *B. subtilis*, and the control effect for Cg and Fp reached 56.2 and 40.00% (Figure 6C). *B. subtilis* reduced the incidence of Cg and Fp by 20 and 10% after control of fresh leaves, where the control effects were 44.44 and 23.08%, respectively. *P. psychrotolerans* reduced the incidence of Cg and Fp by 70 and 50% after control, with control effects of 70 and 61.54%, respectively (Figure 6D).

### 3.5 Expression of endophytic antagonistic bacteria in response to pathogenic fungal infection

*B. subtilis* using primers dnaN-F (5’-GCACTTGCCGCAGATT GA-3′) and dnaN-R (5’-AATGCAAGACGGTGGCTATC-3′). *P. psychrotolerans* using primers 16 s9-F (5’-CTGGCCTTGACAT GCTGAGA-3′) and 16 s9-R (5’-ACCGGCAGTCTCCTTAGAGT-3′). *B. subtilis* expression was the highest after 120 h of infection with Cg and Fp pathogens, followed by a decrease in expression (Figure 6E). *P. psychrotolerans* expression reached two peaks after 24 h and 72 h of infection with Cg pathogens. Expression of Fp was the highest after 168 h of infection (Figure 6F).

### 3.6 Metabolite analysis of two antagonistic bacterial strains

**Figure 7:**
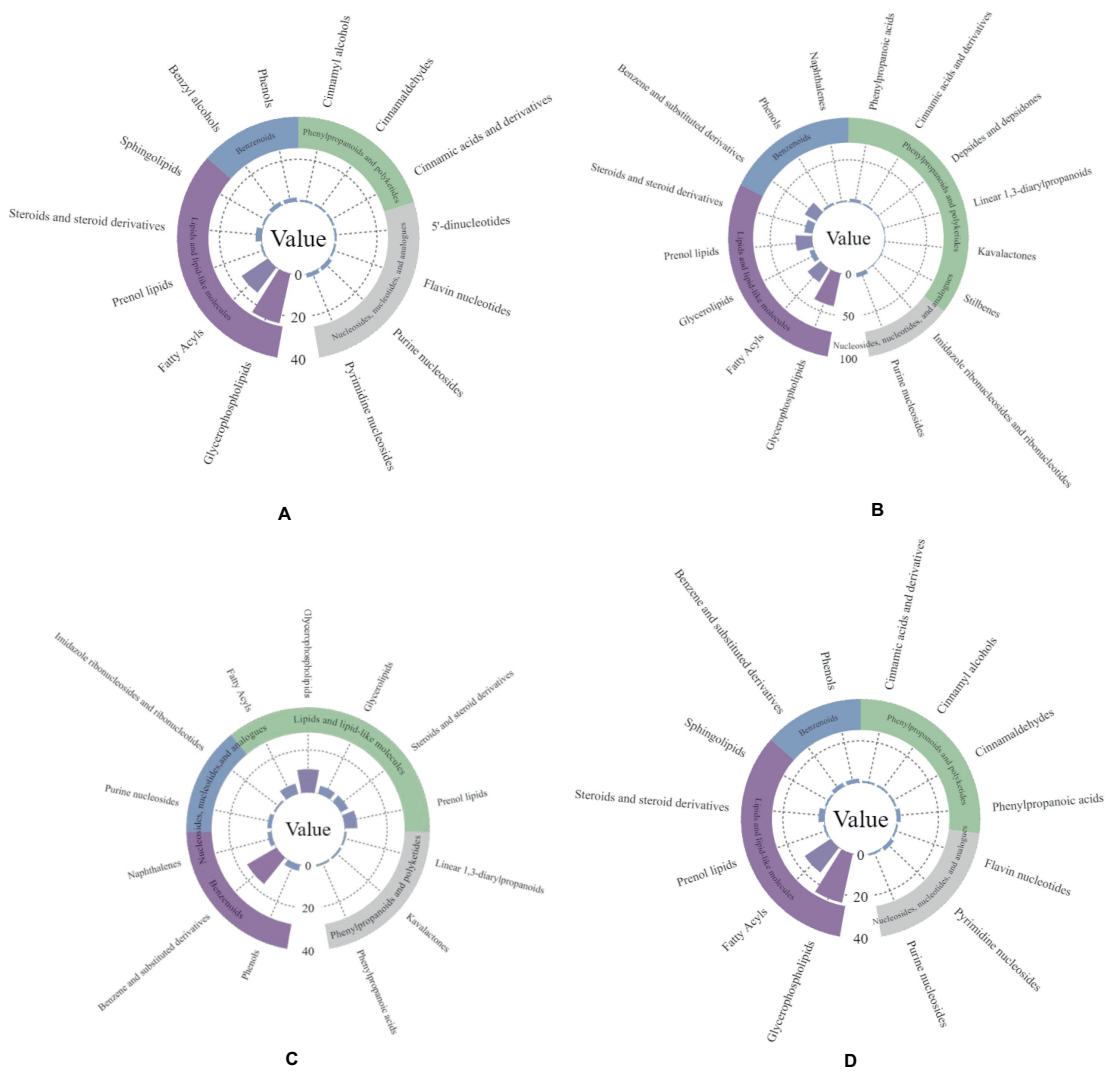
Metabolite enrichment statistics of two antagonistic strains of bacteria. Panel **(A,B)** are *Bacillus subtilis*-positive and *Bacillus subtilis*-negative ion modes; panel **(C,D)** are *Pseudomonas psychrotolerans*-positive and *Pseudomonas psychrotolerans*-negative ion modes.

A large number of metabolites, including lipids, benzenoids, phenylpropanoids, polyketides, nucleosides, and nucleotide metabolites, were enriched for the two antagonistic endophytic bacteria. In addition, metabolites, such as 1-methylnaphthalene, 1,3-butadiene, 2,3-butanediol, and toluene aldehyde, were enriched during pathogenic bacterial infection (Figures 7A–D).

## 4 Discussion

The leaf is an ecologically important part of the plant, and the distribution of endophytic microorganisms and the activity of microbial communities within the leaf significantly impact plant health and growth. The diversity, assembly, and symbiotic network of endophytic microbial communities share a typical response pattern when infected by different pathogenic fungi. This response pattern may be caused by changes in environmental metabolites within the plant and also affects plant disease resistance. Invasion of walnut leaves by two pathogenic fungi increased the network complexity of endophytic fungi, indicating that endophytic fungal community diversity is more sensitive to pathogenic fungal infection than endophytic bacteria. The proportion of bacterial associations was higher in both healthy and diseased plants. In comparison, the proportion of negative associations for bacterial networks and their central taxa was higher in plants than in fungal networks. It is suggested that ecological competition can improve microbiome stability by inhibiting the stability of cooperative associations (Coyte et al., 2015).

The cooperative and competitive nature of microbial species and the modularity of the network can influence the stability of the community (Zhang et al., 2016). Our results suggest that two pathogenic fungi invaded the plant. The host plant may benefit from microbial competition, thus increasing resistance to external stresses (Wagg et al., 2019). Fungal communities were more affected by the pathogenic fungi than bacterial communities, probably due to stronger positive correlations in fungal associations in the network of diseased plants than in the healthy network. The present study showed that endophytic fungal communities are more sensitive to pathogens than bacterial communities, as evidenced by their lower network stability. Previous studies have shown that soil bacterial networks are less stable than fungal networks under drought stress (De Vries et al., 2018). As our samples were sterile histopathogenic seedlings cultured in the same environment, the influence of external factors on the endophytic microbiome of the leaves can be excluded and therefore explains the reliability of the results.

According to previous correlations between host plants and pathogenic fungi, evidence suggests that pathogenic fungal infection promotes the growth and multiplication of certain microorganisms (Seneviratne et al., 2007). Moreover, microorganisms induced to increase due to biotic stress are usually beneficial, and these can enhance host disease resistance in plants (McSpadden Gardener and Weller, 2001; Neal et al., 2012; Santhanam et al., 2015). In the present study, diseased plants were enriched with potentially beneficial bacteria such as *Weisseria* spp., *Bacillus* spp., *Pseudomonas* spp., and *Periplococcus* spp. These beneficial bacteria were also identified as core taxa (i.e., present in all samples and with high relative abundance) in the plant microbiome. Previous studies have shown that *Weissella* spp. and Pseudomonas spp. play an important role in regulating host performance, especially in plant suppression of pathogens (Kim et al., 2018; Bao, 2019). *Bacillus* and *Pseudomonas* have been moderately proven as beneficial bacteria (Moulton and Montie, 1979; Garrity and Ordal, 1995; Sampedro et al., 2015). Some species of the genus *Mortierella* produce antibiotics, and certain isolates are potential antagonists of various plant pathogens and are now used as important indicators of *Fusarium* disease control in ginseng cultivation (Wang et al., 2021). Our results show that host plants can selectively regulate the abundance of some core taxa in the presence of pathogenic bacteria. In addition, some bacterial taxa, such as *Weissella* spp. and *Stenotrophomonas* spp., were enriched in diseased plants and identified as dominant taxa of the pathogen, which were in the co-occurrence network. *Bacillus* spp. and *Pseudomonas* spp. were significantly different after the infection by the two pathogenic fungi. Currently, the elevated relative abundance of these beneficial bacteria facilitates competition with pathogenic fungi for resources and space, such as antimicrobial compounds and population-sensing quenching molecules, which, in turn, inhibit the growth and reproduction and virulent dissemination of pathogenic bacteria (Frey-Klett et al., 2011; Raaijmakers and Mazzola, 2012; Tyc et al., 2017).

Based on the metabolomic analysis, pathogenic fungal infection was found to cause changes in the expression of metabolites within the leaves. The endophytic symbionts could produce antibiotic-like compounds or volatiles that alter the secondary metabolic endogenous environment of the plants and promote direct and indirect plant defense against both pathogenic fungi. Previous studies have shown that many bacteria of the genera *Pseudomonas, Streptomyces, and Bacillus* colonize different plant ecological niches (e.g., interleaf and inter-root) and play an important role in regulating host performance, especially in phytopathogenic suppression (Shweta et al., 2010; Xiong et al., 2017; Wei et al., 2019). In the present study, *Bacillus* spp. and *Pseudomonas* spp. significantly differed from the control after pathogenic bacterial infection. Both genera significantly positively correlated with flavonoid and isoflavonoid differential metabolites. Flavonoids and isoflavonoids exert many physiological functions in plants, are a class of signaling molecules in plant–pathogen interactions, and play a protective role as phytochemicals when plants are subjected to biotic stresses (Dakora et al., 1996). In addition, flavonoids and isoflavonoid metabolites were significantly upregulated after pathogenic fungal infection. The latter metabolites may be involved in the mutualistic relationship between the walnut tree and the two pathogenic fungi, which may play a protective or defensive role against pathogenic fungi in walnuts.

We screened the two endophytic strains with good efficacy against walnut pathogens: *Bacillus subtilis* and cold-tolerant *Pseudomonas* spp. The expression of both strains was upregulated in the plants after inoculation with the pathogenic fungi, which is consistent with the results of our microbiome studies. *B. subtilis* was enriched with a large number of lipid metabolites, benzenes, and purine nucleoside metabolites, all of which increased in expression upon pathogenic fungal infection. This result is in contrast with previous studies that screened the main component as lipopeptides (Huang et al., 2010). However, this contradiction does not imply that there are no fungal inhibitory components similar to lipopeptides as they are rich in antagonistic metabolites, and therefore, more research is needed. *B. subtilis* can use proteins, many sugars, and starch to catabolize tryptophan to form indole. The species can also participate in the purine nucleotide synthesis pathway involved in the host. *P. psychrotolerans* is frequently used in lipase production (Huang et al., 2010) and wastewater treatment (Li et al., 2018) and is enriched in lipid and benzene metabolites. The expression of metabolites such as 1,2-dimyristoylglycerol-3-phosphatidyl-N, N-dimethyl ethanol, and phenylpropanolamine was significantly upregulated. We speculate that these lipid and benzene metabolites may be involved in the action of *P. psychrotolerans* against the two pathogenic fungi, although the specific mechanism of action requires further study.

Several studies claim that the production of certain microbial volatile organic compounds (mVOCs) can be induced or inhibited in specific microbial interactions (Garbeva et al., 2014; Tyc et al., 2015; Piechulla et al., 2017; Kristin et al., 2020). In addition, mVOCs can inhibit pathogenic bacteria and induce plant systemic resistance through direct inhibition (Huang et al., 2012). Indeed, many species of Pseudomonas and Bacillus are used as biocontrol agents of plant pathogens and have been reported to produce volatile organic compounds with antimicrobial activity (Tahir et al., 2017; Xie et al., 2018). Both endophytic antagonistic bacterial strains used in this study were enriched for 1-methylnaphthalene and piperidinium. The production of volatiles such as benzothiazole and 1-methylnaphthalene by *Pseudomonas fluorescens* has been reported to have an inhibitory effect on the tomato pathogen *Ralstonia solanacearum* (Raza et al., 2016). In the present study, we also found 1,3-butadiene and toluene aldehyde in the metabolites of *Bacillus subtilis*. Benzaldehyde and 1,3-butadiene are mVOCs produced by *Bacillus* and have a strong inhibitory activity against *B. subtilis*, the causal agent of cyanobacteria (Tahir et al., 2017). It has been reported that mVOCs alter the transcriptional expression levels of several genes related to motility and pathogenicity, induce systemic resistance in plants, and thus reduce the incidence of wilt (Santoyo, 2021). 1-Methylnaphthalene, 1,3-butadiene, and toluene aldehyde are likely repressors of *B. subtilis* and cold-tolerant *Pseudomonas* spp. screened in this study. However, how the mVOCs of the mesophilic strains modulate the inhibitory effect of the antagonistic bacteria on the pathogenic fungi still needs to be further tested and explored.

## 5 Conclusion

Our results provide novel and relevant insights into the walnut leaf microbiome in response to leaf spot infestation response, with *Colletotrichum gloeosporioides* (Cg treatment) and *Fusarium prolatoratum* (Fp treatment) infesting leaves with both equal expression and species specificity, and the dominant phylum and genus of plant–endophytic bacterial and fungal species were similar but differed only in relative abundance. Pathogenic fungal infections also affected the diversity, network complexity, and stability of endophytic bacterial and fungal communities in walnuts and promoted the enrichment of beneficial bacteria. Our study improves our understanding of the mechanisms by which the endophytic microbiome responds to pathogenic fungal infestation and analyses the leaf metabolome in addition, after pathogenic fungal infestation. In conclusion, the results of this study lay the foundation for an accurate and comprehensive description of microbial community assembly, co-metabolism, and pathogenic fungal interaction patterns within walnut leaves and provide a theoretical basis for microbiome and metabolome regulation of walnut leaf diseases.

## Data availability statement

The datasets presented in this study can be found in the NCBI database, under the accession numbers PRJNA909431 and PRJNA904015.

## Author contributions

ZW: Writing – original draft, Writing – review & editing. LX: Writing – review & editing. XL: Writing – review & editing. RW: Writing – review & editing. JH: Writing – review & editing. AY: Writing – review & editing.
