# Supplementary material for: The endophytic microbiome response patterns of Juglans regia to two pathogenic fungi
Source: Front Microbiol. 2024 Apr 11;15:1378273. doi: 10.3389/fmicb.2024.1378273 (PMC11043491; doi:10.3389/fmicb.2024.1378273)
Supplement: Supplementary file 1 [file Data_Sheet_1.pdf]

Tab S1. Endophyte community correlation network statistics

Community	Group	Number of nodes	Number of edges	Number of communities	Modularity	Postive	Negative	Number of negative associated edges
endophytic bacterial community	CK	79	1227	3	0.526	50.53%	49.47%	607
	Cg	76	369	5	0.496	67.21%	32.79%	121
	Fp	79	327	5	0.549	89.6%	10.4%	34
endophytic fungi community	CK	80	845	5	0.619	70.89%	29.11%	246
	Cg	78	471	6	0.421	76.01%	23.99%	113
	Fp	78	370	6	0.489	94.86%	5.14%	19

Tab.S2 Ck treatment endophytic bacterial community Top80 relative abundance

Nodes	Number of edges	Nodes	Number of edges
Weissella	35	*Lachnospiraceae_NK4A136_group*	17
Lactococcus	34	*Allorhizobium-Neorhizobium-Pararhizobium-Rhizobium*	17
Lactobacillus	33	*Ochrobactrum*	16
Nitrospira	33	*Parabacteroides*	16
Acinetobacter	32	*Campylobacter*	15
Sphingomonas	32	*Romboutsia*	15
Staphylococcus	31	*Enhydrobacter*	14
Bryobacter	31	*Bradyrhizobium*	14
Streptococcus	30	*Delftia*	13
Candidatus_Solibacter	29	*[Ruminococcus]_gnavus_group*	13
Sulfurimonas	29	*Chryseobacterium*	12
Bacteroides	28	*Stenotrophomonas*	12
Escherichia-Shigella	28	*Paracoccus*	11
Faecalibacterium	28	*Christensenellaceae_R-7_group*	10
Agathobacter	28	*Leptotrichia*	9
[Ruminococcus]_torques_group	27	*Tepidimonas*	9
Bifidobacterium	27	*Serratia*	9
Enterococcus	27	*Geobacter*	9
Leuconostoc	26	*Roseburia*	8
Flavisolibacter	26	*Paludibaculum*	8
Pantoea	25	*Ruminococcaceae_UCG-014*	7
Sphingobium	24	*Ramlibacter*	7
Psychrobacter	24	*Aeromonas*	7
Neisseria	24	*Sphingobacterium*	6
Brevundimonas	23	*Ruminiclostridium_9*	6
Ellin6067	23	*Terrimonas*	6
Curtobacterium	22	*Prevotella_9*	5
MND1	22	*Gemmatimonas*	4
Capnocytophaga	21	*Cardiobacterium*	3
Haliangium	21	*Gallibacterium*	3
Rikenellaceae_RC9_gut_group	20	*Fusobacterium*	2
Pseudomonas	18	*Myroides*	2
Comamonas	18	*Devosia*	1
Flavobacterium	18	*Subdoligranulum*	1
Clostridium_sensu_stricto_1	18	*Parasutterella*	1

Note: notes is a point, and the Number of edges is the associated edge of the point. Tab. S3-7 is the same.

Tab.S3 Cg treatment endophytic bacterial community Top80 relative abundance genus level correlation network correlation data statistics

Nodes	Number of edges	Nodes	Number of edges
[Ruminococcus]_torques_group	17	*Nitrospira*	4
Lactococcus	16	*Leuconostoc*	4
Lactobacillus	15	*Leptotrichia*	4
[Ruminococcus]_gnavus_group	15	*Chryseobacterium*	4
Bacteroides	14	*Paludibaculum*	4
Comamonas	13	*Alistipes*	4
Enterococcus	12	*Aeromonas*	3
Faecalibacterium	11	*Terrimonas*	3
Escherichia-Shigella	10	*Weissella*	2
Bryobacter	10	*Sulfurimonas*	2
Ellin6067	10	*Ruminococcaceae_UCG-014*	2
Rikenellaceae_RC9_gut_group	10	*Sphingobium*	2
Sphingomonas	9	*Fusobacterium*	2
Psychrobacter	9	*MND1*	2
Streptococcus	8	*Ramlibacter*	2
Enhydrobacter	8	*Romboutsia*	2
Acinetobacter	7	*Cardiobacterium*	2
Staphylococcus	7	*Tepidimonas*	2
Pantoea	7	*Subdoligranulum*	2
Bacillus	7	*Lachnoclostridium*	2
Candidatus_Solibacter	7	*Gallibacterium*	2
Pseudomonas	7	*Bifidobacterium*	1
Neisseria	7	*Brevundimonas*	1
Haliangium	7	*Curtobacterium*	1
Allorhizobium-Neorhizobium-Pararhizobium-Rhizobium	7	*Sphingobacterium*	1
Paenibacillus	6	*Chryseobacterium*	1
Flavisolibacter	6	*Ruminiclostridium_9*	1
Clostridium_sensu_stricto_1	6	*Parasutterella*	1
Flavobacterium	6	*UTBCD1*	1
Agathobacter	6	*Geobacter*	1
Roseburia	6	*Prevotella_9*	1
Campylobacter	6	*Myroides*	1
Capnocytophaga	5	*Ferruginibacter*	1
Blautia	5	*Pajaroellobacter*	1

Tab.S4 Fp treatment endophytic bacterial community Top80 relative abundance genus level correlation network correlation data statistics

Nodes	Number of edges	Nodes	Number of edges
Staphylococcus	14	*Capnocytophaga*	4
Lactococcus	12	*Rikenellaceae_RC9_gut_group*	4
[Ruminococcus]_torques_group	11	*Agathobacter*	4
Bryobacter	11	*Campylobacter*	4
Curtobacterium	10	*Desulfovibrio*	4
Candidatus_Solibacter	10	*Psychrobacter*	3
Enterococcus	10	*Enhydrobacter*	3
Lactobacillus	9	*Pseudolabrys*	3
Nitrospira	9	*Chryseobacterium*	3
Clostridium_sensu_stricto_1	9	*Ramlibacter*	3
Weissella	8	*Delftia*	3
Streptococcus	8	*Cardiobacterium*	3
Comamonas	8	*Paludibaculum*	3
Bacteroides	7	*Blautia*	2
Pantoea	7	*Sphingobacterium*	2
Pseudomonas	7	*MND1*	2
Flavisolibacter	7	*Bradyrhizobium*	2
Flavobacterium	7	*Stenotrophomonas*	2
Haliangium	7	*Roseburia*	2
Bacillus	7	*Aeromonas*	2
Escherichia-Shigella	6	*Ruminiclostridium_9*	2
Faecalibacterium	6	*[Ruminococcus]_gnavus_group*	2
Neisseria	6	*Alistipes*	2
Ellin6067	6	*Myroides*	2
Sphingomonas	5	*Ruminococcaceae_UCG-014*	1
Leuconostoc	5	*Leptotrichia*	1
Sphingobium	5	*Geobacter*	1
Lachnospiraceae_NK4A136_group	5	*Gallibacterium*	1
Allorhizobium-Neorhizobium-Pararhizobium-Rhizobium	5	*UTBCD1*	1
Parabacteroides	5	*Terrimonas*	1
Acinetobacter	4	*Paracoccus*	1
Sulfurimonas	4	*Lysobacter*	1
Bifidobacterium	4	*Rahnella*	1

Tab.S5 Ck treatment endophytic fungi community Top80 relative abundance genus level correlation network correlation data statistics

Nodes	Number of edges	Nodes	Number of edges
Filobasidium	29	*Monocillium*	10
Vishniacozyma	28	*Clitocybe*	10
Occultifur	27	*Trichoderma*	9
Cystofilobasidium	26	*Chaetomium*	9
Penicillium	25	*Pyrenochaetopsis*	9
Grifola	24	*Geopora*	8
Udeniomyces	23	*Cephalotrichum*	8
Colletotrichum	22	*Malassezia*	7
Naganishia	22	*Echria*	7
Pleurotus	21	*Gaeumannomyces*	7
Alternaria	20	*Tremella*	7
Schizothecium	20	*Byssochlamys*	6
Epicoccum	20	*Kurtzmaniella*	6
Mortierella	19	*Lecanicillium*	6
Trechispora	19	*Cyphellophora*	5
Talaromyces	18	*Cutaneotrichosporon*	5
Leptobacillium	18	*Inocybe*	5
Exophiala	18	*Preussia*	5
Cylindrocladiella	17	*Sampaiozyma*	4
Fusarium	16	*Wickerhamomyces*	4
Enterocarpus	16	*Periconia*	4
Candida	16	*Chaetosphaeria*	4
Aspergillus	15	*Didymosphaeria*	3
Cladorrhinum	15	*Phialocephala*	3
Monascus	15	*Acremonium*	3
Cladosporium	14	*Coniochaeta*	3
Botryotrichum	14	*Starmerella*	2
Aureobasidium	13	*Wickerhamiella*	2
Kazachstania	13	*Kernia*	2
Neocucurbitaria	13	*Phaeoisaria*	2
Humicola	13	*Geminibasidium*	2
Sarocladium	12	*Condenascus*	2
Sagenomella	12	*Sporobolomyces*	1
Plectosphaerella	11	*Symmetrospora*	1
Ustilaginoidea	11	*Stropharia*	1
Ascochyta	11	*Zygosaccharomyces*	1
Trichomerium	11	*Clarireedia*	1
Gibellulopsis	10		

Tab.S6 Cg treatment endophytic fungi community Top80 relative abundance genus level correlation network correlation data statistics

Nodes	Number of edges	Nodes	Number of edges
Penicillium	25	*Epicoccum*	5
Occultifur	24	*Pseudeurotium*	5
Mortierella	21	*Panaeolus*	5
Aureobasidium	20	*Colletotrichum*	4
Botryotrichum	20	*Cladosporium*	4
Talaromyces	18	*Schizothecium*	4
Kazachstania	18	*Preussia*	4
Chaetomium	17	*Exophiala*	4
Vishniacozyma	14	*Naganishia*	3
Cephalotrichum	14	*Trechispora*	3
Lecanicillium	14	*Inocybe*	3
Trichoderma	12	*Cyphellophora*	3
Grifola	12	*Scleroderma*	3
Pyrenochaetopsis	12	*Chloridium*	3
Pleurotus	11	*Myrmecridium*	3
Humicola	11	*Fusarium*	2
Ustilaginoidea	11	*Alternaria*	2
Conocybe	10	*Gibellulopsis*	2
Nigrospora	9	*Acremonium*	2
Leptobacillium	9	*Kurtzmaniella*	2
Echria	9	*Byssochlamys*	2
Enterocarpus	8	*Geopora*	2
Sagenomella	8	*Candida*	2
Boothiomyces	8	*Paraphaeosphaeria*	2
Thermoascus	8	*Thermomyces*	2
Thelebolus	8	*Dactylella*	2
Filobasidium	7	*Aspergillus*	1
Cystofilobasidium	7	*Cladorrhinum*	1
Sarocladium	6	*Achroiostachys*	1
Udeniomyces	6	*Sampaiozyma*	1
Clonostachys	6	*Kernia*	1
Simplicillium	6	*Cutaneotrichosporon*	1
Plectosphaerella	5	*Monascus*	1

Tab.S7 Fp treatment endophytic fungi community Top80 relative abundance genus level correlation network correlation data statistics

Nodes	Number of edges	Nodes	Number of edges
Alternaria	22	*Ilyonectria*	4
Cladosporium	20	*Purpureocillium*	3
Colletotrichum	15	*Vishniacozyma*	3
Penicillium	15	*Simplicillium*	3
Epicoccum	13	*Wickerhamomyces*	3
Malassezia	13	*Phialocephala*	3
Leptobacillium	12	*Geopora*	3
Botryotrichum	11	*Monascus*	3
Pyrenochaetopsis	10	*Inocybe*	3
Enterocarpus	10	*Sampaiozyma*	3
Aspergillus	9	*Pleurotus*	2
Sarocladium	9	*Grifola*	2
Talaromyces	9	*Gibellulopsis*	2
Aureobasidium	8	*Naganishia*	2
Kazachstania	8	*Chloridium*	2
Schizothecium	8	*Agrocybe*	2
Condenascus	8	*Chaetomium*	2
Boothiomyces	7	*Zygosaccharomyces*	2
Cyphellophora	7	*Preussia*	2
Sagenomella	6	*Acremonium*	2
Trechispora	6	*Humicola*	2
Exophiala	6	*Arxotrichum*	2
Cylindrocladiella	6	*Monocillium*	2
Ustilaginoidea	6	*Chaetosphaeria*	2
Filobasidium	5	*Trichoderma*	1
Gliocladiopsis	5	*Conocybe*	1
Plectosphaerella	5	*Udeniomyces*	1
Lecanicillium	5	*Clonostachys*	1
Cephalotrichum	5	*Kurtzmaniella*	1
Cladorrhinum	5	*Byssochlamys*	1
Echria	5	*Scytalidium*	1
Candida	5	*Botryotinia*	1
Nigrospora	4	*Neocucurbitaria*	1

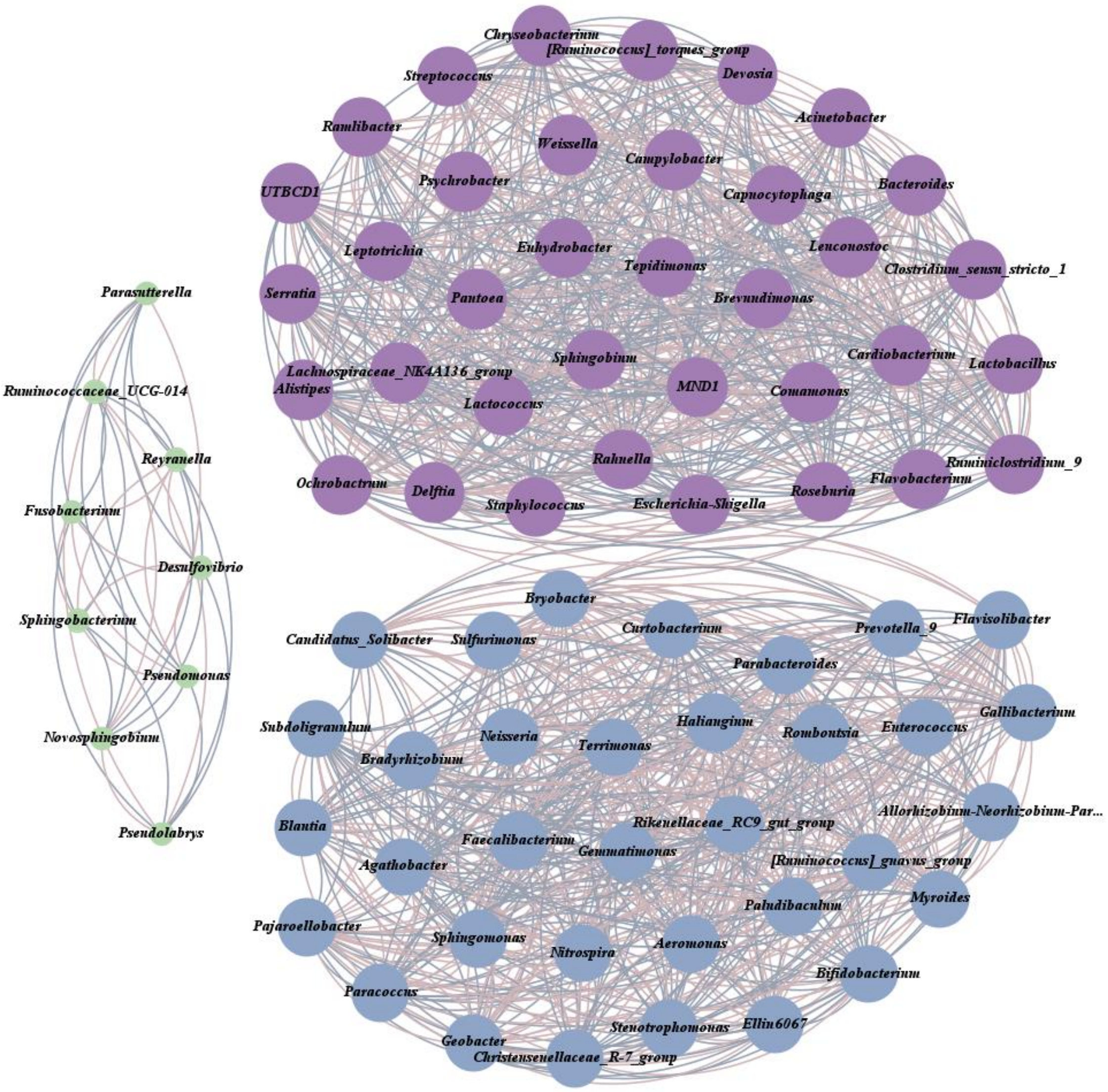

Fig.S1

Fig.S1 CK endophytic bacteria top80 genus correlation network .

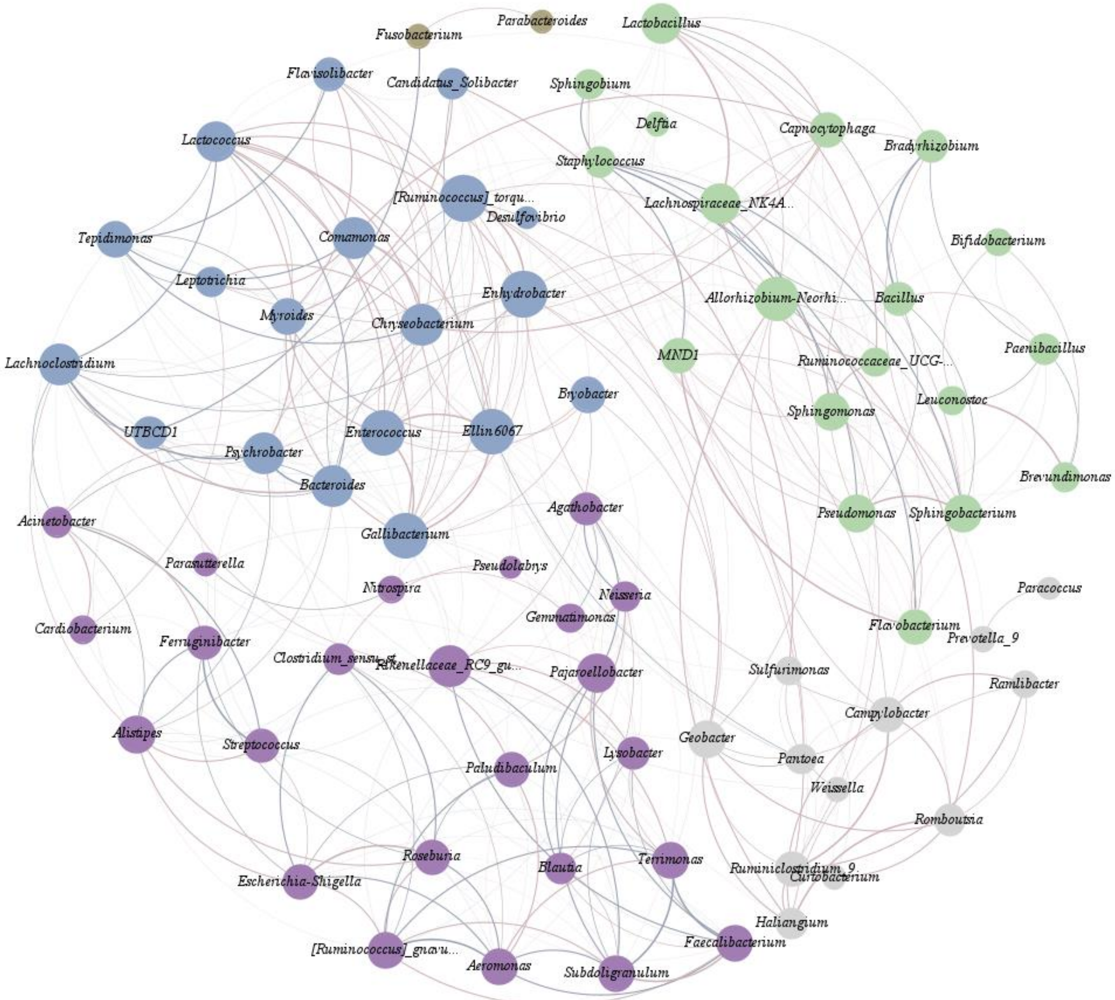

Fig.S2

Fig.S2 Cg treat endophytic bacteria top80 genus correlation network diagram

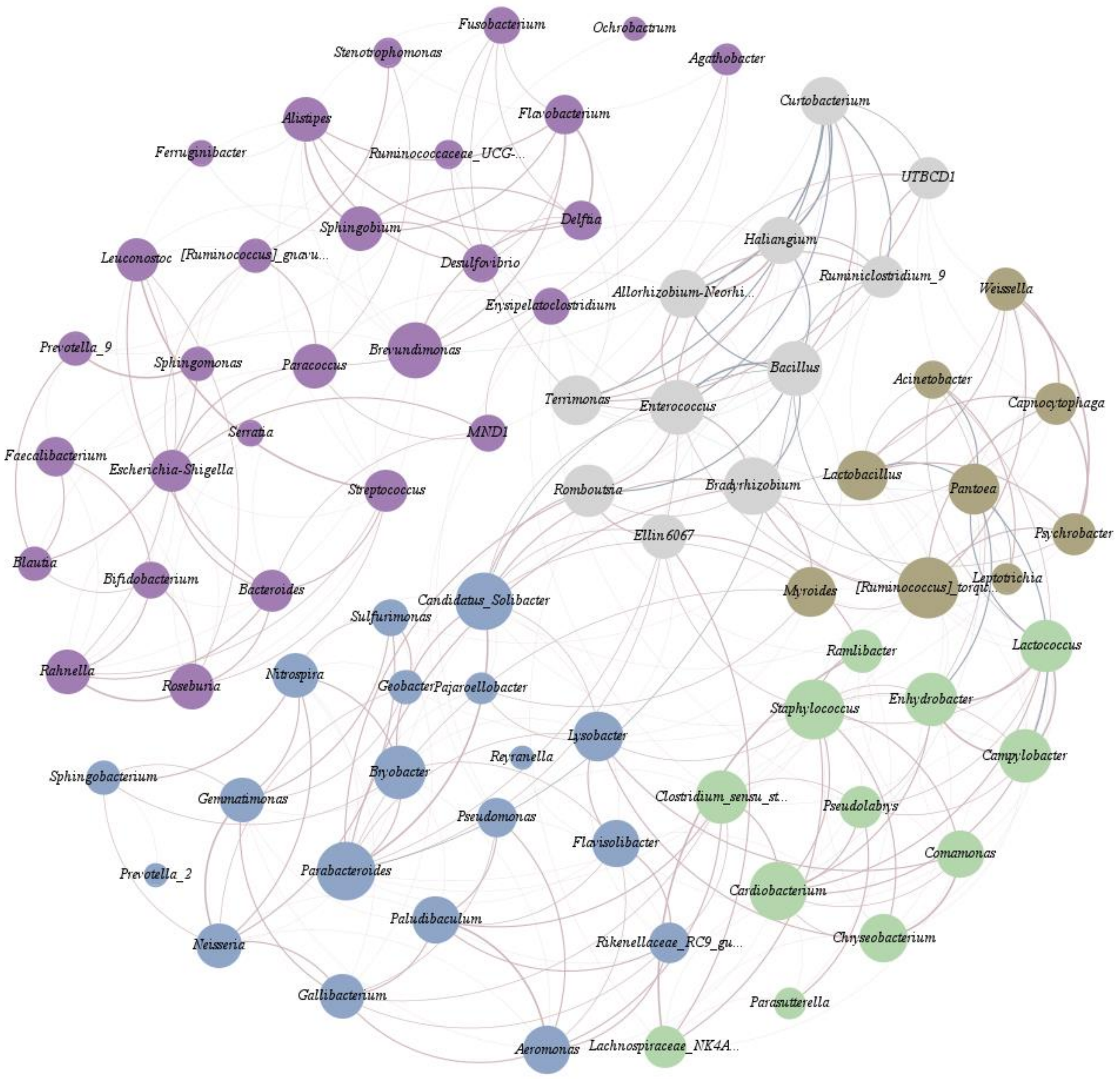

Fig.S3

Fig.S3 Fp treat endophytic bacteria top80 genus correlation network diagram

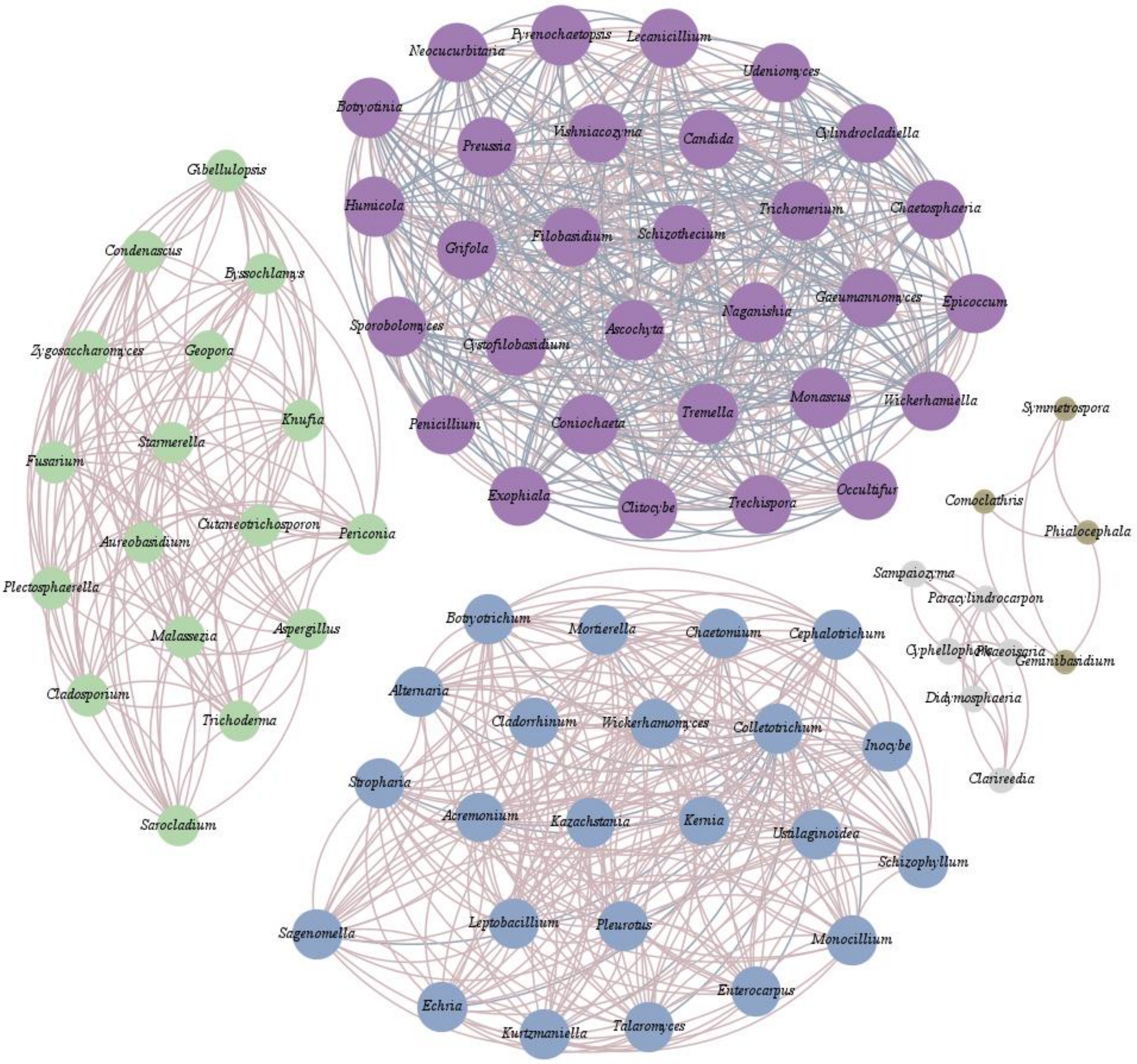

Fig.S4

Fig.S4 Ck treat endophytic fungi top80 genus correlation network diagram

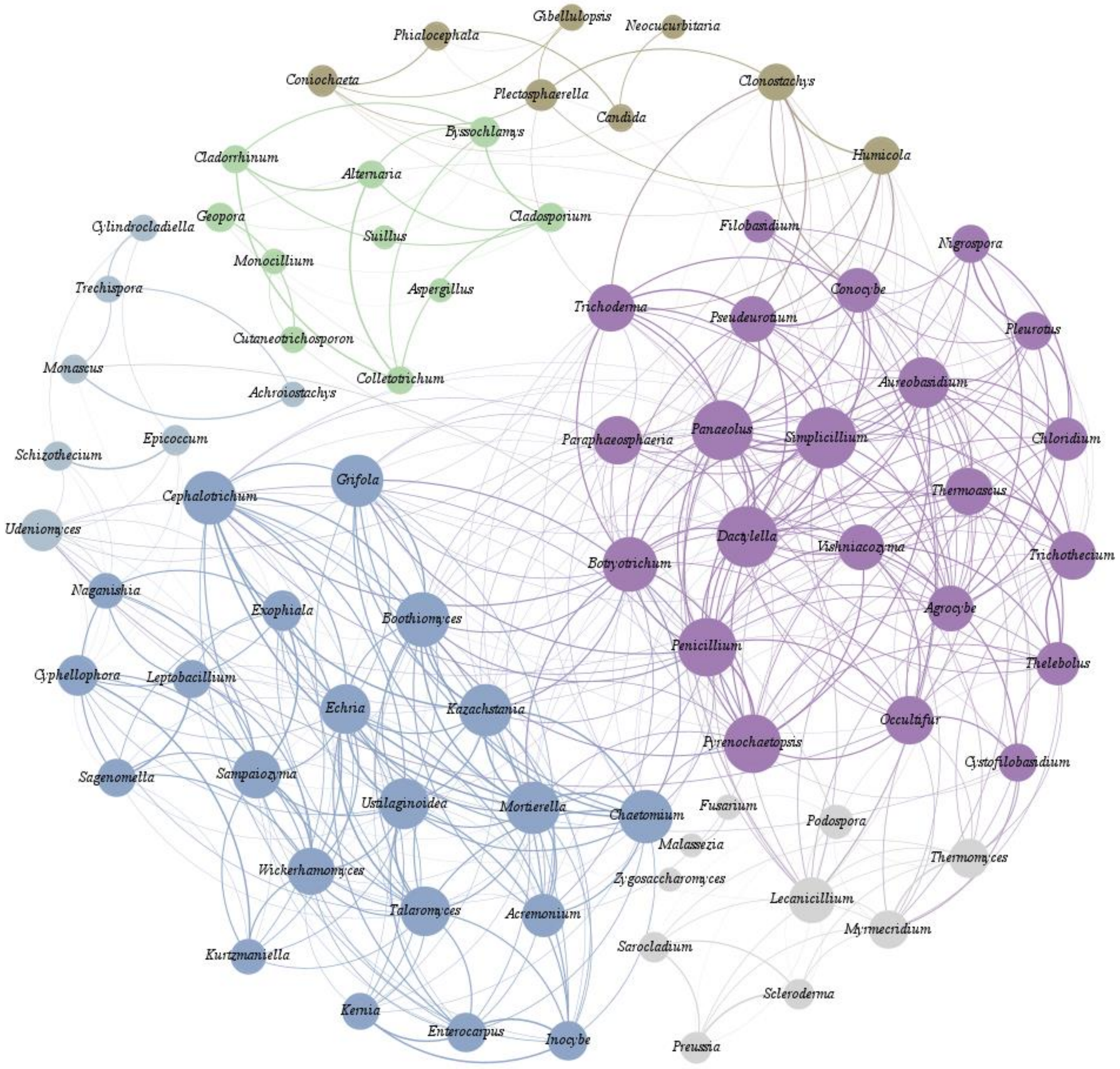

Fig.S5

Fig.S5 Cg treat endophytic fungi top80 genus correlation network diagram

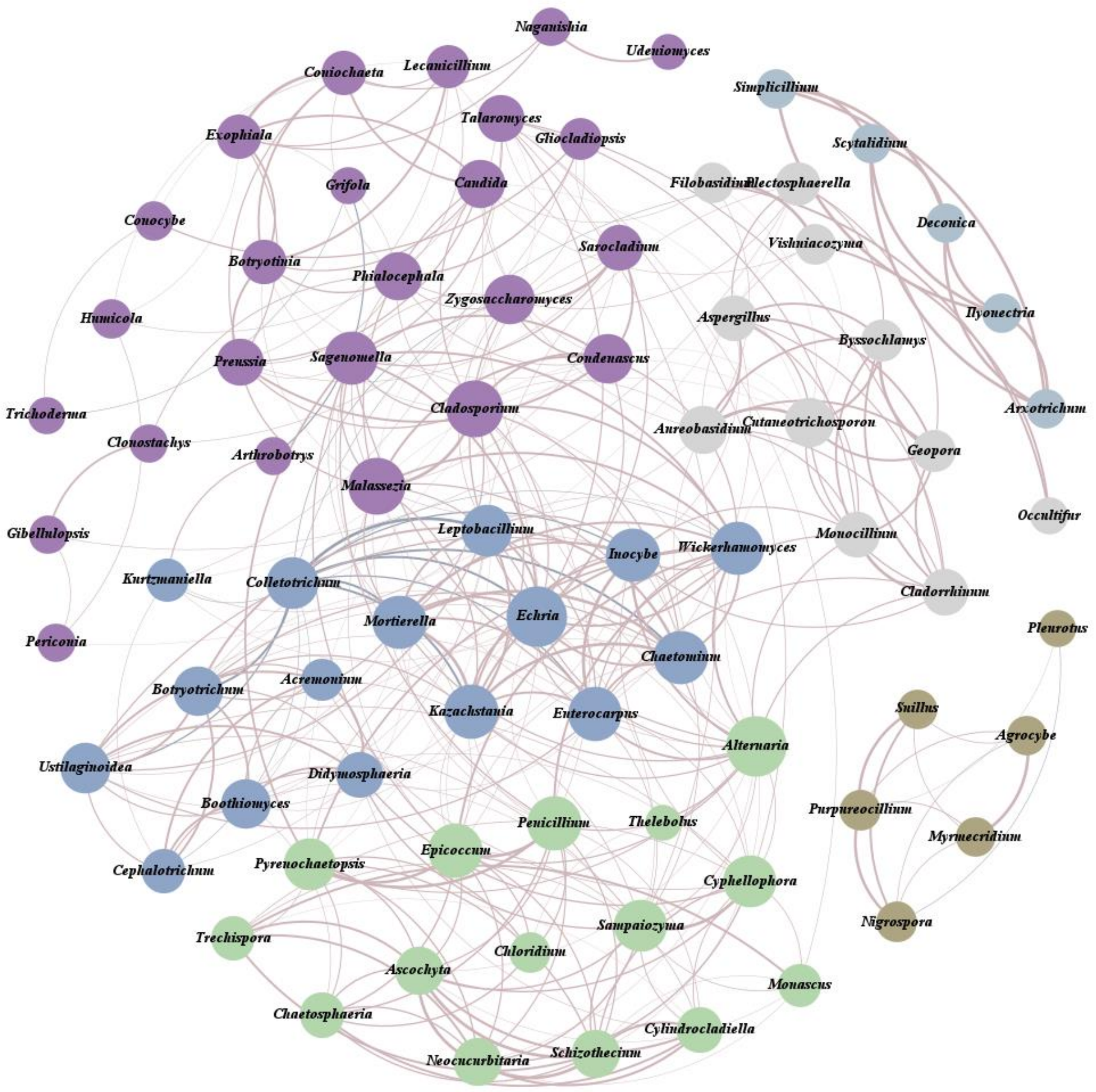

Fig.S6

Fig.S5 Fp treat endophytic fungi top80 genus correlation network diagram
